# Application of Deep Reinforcement Learning to NS-SHAFT Game Signal Control

**DOI:** 10.3390/s22145265

**Published:** 2022-07-14

**Authors:** Ching-Lung Chang, Shuo-Tsung Chen, Po-Yu Lin, Chuan-Yu Chang

**Affiliations:** 1 Department of Computer Science and Information Engineering, National Yunlin University of Science and Technology, Douliu 640301, Taiwan; chang@yuntech.edu.tw (C.-L.C.); shough33@gmail.com (P.-Y.L.); chuanyu@yuntech.edu.tw (C.-Y.C.); 2 Intelligence Recognition Industry Service Research Center (IR-IS Research Center), National Yunlin University of Science and Technology, Douliu 640301, Taiwan; 3 Department of Applied Mathematics, Tunghai University, Taichung 40704, Taiwan; 4 Department of Industrial and Business Management, Chang Gung University, Taoyuan 333, Taiwan

**Keywords:** reinforcement learning (RL), game, real-time, Deep Q-Network (DQN), deep learning, NS-SHAFT

## Abstract

Reinforcement learning (RL) with both exploration and exploit abilities is applied to games to demonstrate that it can surpass human performance. This paper mainly applies Deep Q-Network (DQN), which combines reinforcement learning and deep learning to the real-time action response of NS-SHAFT game with Cheat Engine as the API of game information autonomously. Based on a personal computer, we build an experimental learning environment that automatically captures the NS-SHAFT’s frame, which is provided to DQN to decide the action of moving left, moving right, or stay in same location, survey different parameters: such as the sample frequency, different reward function, and batch size, etc. The experiment found that the relevant parameter settings have a certain degree of influence on the DQN learning effect. Moreover, we use Cheat Engine as the API of NS-SHAFT game information to locate the relevant values in the NS-SHAFT game, and then read the relevant values to achieve the operation of the overall experimental platform and the calculation of Reward. Accordingly, we successfully establish an instant learning environment and instant game training for the NS-SHAFT game.

## 1. Introduction

Machine learning is the main technology for the development of contemporary artificial intelligence [1,2,3,4,5,6,7,8,9,10]. With the improvement of hardware equipment, people are reinvesting in machine learning research. The most striking thing is that the AlphaGo [11] developed by the DeepMind team combines the two major technologies of machine learning, deep Learning [12] and reinforcement Learning [13], to defeat the Go world champion in March 2016.

In 2013, the DeepMind team was the first to propose a combination of reinforcement learning (RL) and deep learning (DL), called Deep Q-Network (DQN) [14], which was successfully practiced on Atari games and compared with other methods. By comparison, six of the seven games have scored more than other methods, and several have exceeded the level of professional players. Different from the previous method of manually extracting features, they proposed a model that combines reinforcement learning and deep learning. The gameplay strategy can be learned only through the original game screen as input, and it can be learned in other games without adjusting any neural network architecture and parameters, demonstrating its versatility. However, they used the same network to estimate the target value and the predicted value so that there was a great correlation between the two, which led to the problem of unstable training.

Based on the 2013 method, DeepMind added an improved method TargetNetwork [15] in 2015 which aims to solve the shortcomings of the unstable Q value of prediction. This method expands the experimental games from 7 to 49, and in many games, it not only surpasses the past methods, but also surpasses professional players. At the same time, it is also found that DQN can achieve very good learning effects in some pure scoring games, such as Breakout, and for games that require specific steps to continue to break through, such as Montezuma’s revenge, it performs very badly. In other words, for different types of games, DQN has very different effects.

After DeepMind solved the problem of unstable Q value, it found that Q-Learning itself has the shortcoming of overestimating. The problem is that Q-Learning adopts a greedy method when selecting actions, so that when selecting or estimating the value of an action, if the value of the sub-optimal action is overestimated during the training process, and thus exceeds the value of the optimal action, it will cause the entire learning process to never find the optimal action. Based on the Double Q-Learning proposed by Hasselt [16] in the Q-Learning experiment in 2010, DeepMind proposed a method of improving overestimation in 2015 called Double DQN (DDQN) [17] to separate the selection and estimation actions. Since the DQN with Target Network has two networks at the same time, there is no need to make major adjustments to the overall architecture. Dong et al. [18] designed a simple reinforcement learning (RL) agent that implements an optimistic version of Q-learning and establish through regret analysis that this agent can operate with some level of competence in any environment. They considered a general agent-environment interface and provide a novel agent design and analysis based on the concepts from the literature on provably efficient RL.

Based on the rapid development of current hardware equipment and software technology, this paper mainly combines reinforcement learning and deep learning technology, called Deep Q-Network (DQN), to process real-time action response of NS-SHAFT game with analysis of the game information by Cheat Engine. The detail is as follows. First of all, we build a real-time learning environment in a personal computer to automatically capture the screen of NS-SHAFT and then provide DQN to decide the action of moving left, moving right, or stay in same location, survey different parameters: such as the sample frequency, different reward function, and batch size etc. Next, we discuss the impact of different parameters in the DQN environment, such as sample frequency, reward function, batch size, and other parameters on learning effectiveness. Moreover, because NS-SHAFT does not provide any way to directly obtain game internal information and does not provide any game information API, we use open source software, Cheat Engine, to analyze game information to obtain relevant numerical memory addresses and required game information of Reward Function to achieve real-time training for NS-SHAFT game. Experimental results not only show that the relevant parameter settings have a certain degree of influence on the learning effect of DQN, but also successfully establish an instant learning environment and instant game training for the NS-SHAFT game.

The reset of this paper is organized as follows. Section 2 reviews preliminaries and background. In Section 3, we introduce the proposed method and system. Section 4 shows the simulation results. Finally, Section 5 concludes this work.

## 2. Preliminaries and Background

In this section, we review some preliminaries and background for later use.

### 2.1. Introduction to NS-SHAFT

**Figure 1:**
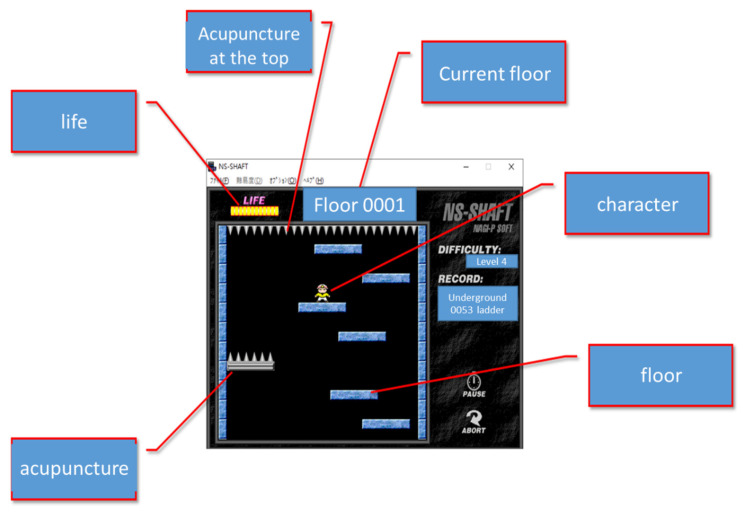
NS-SHAFT screen.

NS-SHAFT is a PC game [17]. As shown in Figure 1, the player moves the character left and right in the game to increase the current number of floors as much as possible. The game provides a choice of three difficulty levels. During the game, when the number of floors becomes larger, the scrolling speed of the game will increase and the proportion of needle sticks will also increase, making the operation thinking time shorter and shorter. The game ends when the character falls to the bottom of the game screen. The character in the game has 12 points of life. When the character touches the fixed needle or the floor, the character will lose 5 points of life. When the character’s life returns to 0, the game also ends. However, the character will restore 1 point of life when it’s life is less than 12 points and land on the normal floor.

### 2.2. Reinforcement Learning

**Figure 2:**
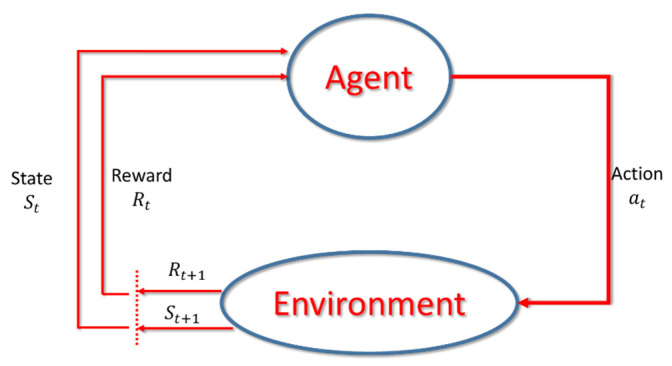
Diagram of reinforcement learning.

Reinforcement Learning is a major branch in the field of machine learning, focusing on how agents can deliver actions to the environment and find out a policy to maximize their future benefits (or reward) by trial-and-error where the method by which the agent selects the action from the environment state (*S*) is called the policy [19,20,21]. The detail is as follows. As shown in Figure 2, the Agent will use the known policy or randomly select the action Action $a_{t}$ according to the current state *S_t_*. At the time, the state *S_t_* will transfer to the new state *S_t+1_*, and get a reward *R_t_*.

In this learning process, when the correct action is taken, the system will give a positive reward, if it is a wrong action, it will give a negative reward. With the reward value, the Agent can learn the best action strategy to get the maximum reward. Generally speaking, we would like to choose the action that will bring the greatest reward every time. This strategy is called greedy. In other words, greedy is a way of choosing the best choice at every step to expect the best result. However, this method has the problem of falling into a local optimal solution. It is usually solved by using the *ε*-greedy algorithm which has a certain probability *ε* for randomly choosing actions.

In reinforcement learning, if only the reward value can only reflect the immediate situation in a certain state, it cannot accurately reflect the actual value from a certain state to the end of the round. In other words, the purpose is to evaluate if a state has the maximum value after taking an action instead of the current maximum reward value. Accordingly, a value function *V*(*S*) is defined by

(1) $$V^{\pi }(S)=E_{\pi }(R_{t}|S_{t}=S)=E_{\pi }(\sum_{i=0}^{\infty }\lambda^{i}r_{t+i+1}|S_{t}=S)$$

where $V^{\pi }(S)$ is the total rewards obtained from state *S* following policy π; $E_{\pi }$ denotes the total rewards obtained under the policy π; $R_{t}$ denotes the reward accumulated in state *s* at time *t*; $S_{t}$ denotes the state at time *t, t* = 1,2,3,…; and λ, 0≤λ≤1, denotes the discount rate used to avoid excessive states leading to infinite total rewards for each strategy.

Value function is used to evaluate the value of each state, but, in fact, each state has different actions. We need to evaluate all the actions in the same state separately to obtain the value of each action in that state. Therefore, the action and state are regarded as a combination, and the action value function *Q*(*s*, *a*) is derived.

(2) $$Q^{\pi }(S,a)=E_{\pi }(R_{t}|S_{t}=S,a_{t}=a)=E_{\pi }(\sum_{i=0}^{\infty }\lambda^{i}r_{t+i+1}|S_{t}=S,a_{t}=a)$$

where $Q^{\pi }(S,a)$ is the total rewards obtained from that state *S* select action *a* and follow policy π; $E_{\pi }$ denotes the total rewards obtained under the policy π; $R_{t}$ denotes the reward accumulated in state *s* with selection of action *a* at time *t*; $S_{t}$ denotes the state at time *t, t* = 1,2,3, …; and λ, 0≤λ≤1, denotes the discount rate used to avoid excessive states leading to infinite total rewards for each strategy.

### 2.3. Q-Learning

The purpose of Q-Learning is to record the entire learning process [22]. All actions taken under the state and rewards will be fully recorded to form a Q-Table as shown in Table 1. From this table, the agent can know which action will get the greatest reward. The detail is shown in Algorithm 1. At the beginning of the algorithm, the initial value of *Q* is arbitrarily set (determined by the developer), and training is performed for each step in each episode. After the action (*a*) is given to the state (*s*), the reward (*r*) and the new state (*s*’) can be obtained, and then the value Q(*s*, *a*) is updated corresponding to the state *s* and action *a* in the Q-Table. In the update formula, Q*(s, a)* is the benefit obtained by taking action *a* in the *s* state, r is the reward value obtained by taking action *a* in the current state *s*, *α* is the learning rate, and γ is the discount factor (Discount factor). When updating, the algorithm will care about the current reward (*r*) and the reward in memory $\underset{a^{\prime }}{\max}\left\{Q\left(s^{\prime },a^{\prime }\right)\right\}$. The reward in the memory represents the maximum reward value that the new state *s’* can give. If the agent takes an action in the past *s*’ to obtain the reward value, this formula can make it learn the news early. When entering *s* again, choose the correct action to continue entering *s*’ in order to get rewards. Therefore, the larger γ the agent will pay more attention to the past experience, otherwise the agent will pay attention to the current reward *r*. The algorithm is summarized as
**Algorithm 1.**Q-Learning.
Initialize Q(*s*, *a*) arbitrarilyRepeat (for each episode):   Initialize *s*   Repeat (for each step of episode):    Take action *a*, observe *r*, *s*^’    Chose *a* from *s* using policy derived from Q (e.g., ε-greedy)    Q(*s*, *a*) ← Q(*s*, *a*) + α[*r* + γ〖max〗_*a*’Q(*s*^’, *a*^’) − Q(*s*, *a*)]    *s* ← *s*^’; until *s* is terminal


### 2.4. Deep Q-Network

In order to solve the shortcomings of Q-Learning in storing Q-Tables due to the excessive number of states and actions, the DeepMind team proposed Deep Q-Network [14] and verified it on the Arcade Learning Environment (ALE) [23] environment. Deep Q-Network directly uses the entire game screen as the input of the model, so that the model learns which features to obtain through a lot of training. For this reason, there is no need to consider which features the model finally obtains, as long as the model can output the maximum reward value in various states.

In addition, the pixels of the game screen are too large, and each pixel has 256^3^ possibilities, but, in fact, there is a correlation between the pixels, and it is not necessary to treat each pixel as an input. Convolutional neural network (CNN) [24,25] can solve the problem at this time. Therefore, Deep Q-Network has a CNN model that can handle the input of the game screen, which is composed of one or more convolutional layers and fully connected layers. The model finally outputs the *Q* value of each action.

Finally, in order to make the model more accurate prediction, a lot of data is used in the model to iterate. The data can be obtained through the continuous game process. The algorithm uses the Experience Replay technology [26] to store the game process data in One is called replay memory. The stored data include state, action, reward, and {state}^’ (The next state entered after an action is taken). In the training process, a certain number of samples are selected from the replay memory as the model input for algorithm iteration. The overall algorithm is shown below.

Step 1. Initialize a space D with Size *N* for storing past experience.

Step 2. Randomly initialize the *Q* of the neural network, and then play M games, each end of the game represents an episode.

Step 3. At the beginning of each episode, set the game screen as the state (*S*_1_), and preprocess this game screen into $\emptyset_{1}$ = ∅($s_{1}$).

Step 4. Each step in the game randomly selects the action *a_t_* with probability *ϵ*, and the 1-*ϵ* probability uses the optimal action *a_t_* predicted by the neural network *Q*.

Step 5. Following the Step 4, the action *a*_t_ of each $\emptyset_{t}=$∅($s_{t}$) has reward *r_t_* and next state *S_t_*_+1_. Perform the same preprocessing on state *S_t_*_+1_ to get $\emptyset_{t+1}$= ∅($s_{t+1}$) and ten save the relevant information of this state transition ($\emptyset_{t}$, *a*_t_, *r_t_*, ∅*_t_*_+1_) to replay memory.

Step 6. Randomly select a certain number of transitions information from the replay memory, and calculate the *Q* value according to the formula.

- When $\emptyset_{j+1}$ is over (that is, taking action *a_j_* in state $\emptyset_{j}$ leads to the end of the game), make the target value to be *y_j_ = r_j_*;
- When $\emptyset_{j+1}$ is not over yet (that is, an action *a_j_* is taken in the state $\emptyset_{j}$ so that the game is not over), the target value is *y_j_* = *r_j_* + $\underset{a^{\prime }}{\gamma \max}\left\{Q\left(\phi_{j+1},a^{\prime };\theta \right)\right\}$ where *θ* stands for neural network.

Step 7. Finally, mini-batch gradient descent is used to reduce the loss function to improve the neural network.

### 2.5. Deep Q-Network with Target Network

In 2015, DeepMind added a method, Target Network, to improve Deep Q-Network. It mainly calculates the error between TD target and the current estimated value Q(*s, a*), which is defined as follows:

(3) $$\Delta w=\alpha \left\{\left[R+\gamma \underset{a}{\max}\hat{Q}(s^{\prime },a,w)\right]-\hat{Q}(s,a,w)\right\}$$

where ∆w represents the weight to be updated for this difference, *α* is the learning rate, $R+\gamma \underset{a}{\max}\hat{Q}(s^{\prime },a,w)$ is the TD target, and $\hat{Q}(s,a,w)$ represents the estimated *Q* value of the current state.

In fact, we don’t know the real TD target. We see that the TD target is only a reward for taking the action in this state, plus the highest Q worth discount for the next state. The problem is that we use the same network (weight) To estimate the TD target and Q value, so there is a great correlation between the TD target and the network (w) we are changing, which means that in each step of training, the Q value will move, but the target value (TD target) will also move. It is like chasing a moving target, which makes the training shock.

Compared with 2013, the 2015 version of DQN only modifies the calculation of the target value (TD target) and keeps the rest the same to get better results. After that, some scholars still made improvements to DQN. For example, Schaul et al. [27] proposed Prioritized experience replay by extracting samples in a non-random manner, or Wang, Z. et al. [28] modified the neural network in a small amount. In 2018, Horgan et al. [29] used a distributed method to obtain data to improve stability. The above-mentioned studies all improved DQN step by step and enhanced its effectiveness.

### 2.6. Related Work

In 2013, the DeepMind team is the first to propose an algorithm that combines Reinforcement learning (RL) and Deep learning (DL) called Deep Q-Network (DQN) [14] and successfully practiced it on Atari games. The model combining reinforcement learning and deep learning proposed in this paper is different from the previous method of manually extracting features, that is, the game strategy can be learned only by using the original game screen as input, and without adjusting any neural network architecture and parameters. Learning from other games shows its versatility. They compared the results of the practice with random, past methods [13,19] and human battle, as shown in Table 2. Six of the seven games outperformed the random and the past methods [13,19], and several outperformed professional players. In 2015, DeepMind improved the instability of *Q* value in DQN by adding the method TargetNetwork [15] and then enhanced the performance as shown in the last row of Table 2.

## 3. Proposed Method

**Figure 3:**
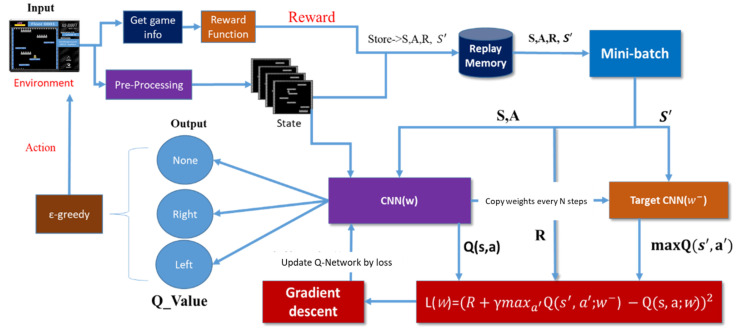
The proposed architecture.

This section introduces the proposed architecture as shown in Figure 3. The detail of this architecture is explained in Section 3.1, Section 3.2, Section 3.3.

### 3.1. Image Pre-Processing

**Figure 4:**
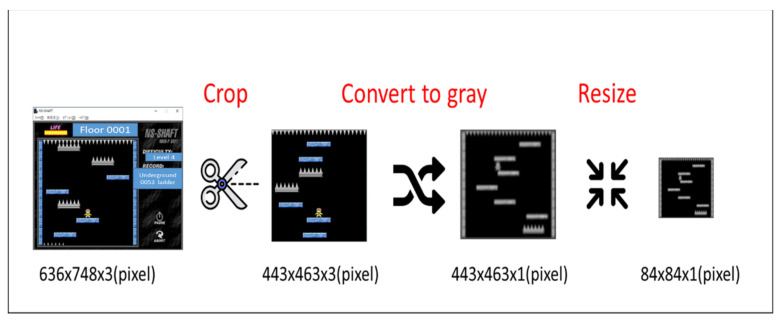
The flowchart of image pre-processing.

In the first step of the proposed architecture, we pre-process original images of NS-SHAFT. When we get the game screen from the game environment, as shown in Figure 4, we can cut the part that does not affect the game judgment so that the entire game screen does not need to be used as input. Therefore, we cut the original game screen size (636 × 478 × 3) Pixels to (443 × 463 × 3) Pixels. Since the color has no meaning for the game execution, we converted the RGB image into a grayscale image to reduce the data dimension, that is, 443 × 463 × 3 Pixels is reduced to 443 × 463 × 1 Pixels. Moreover, we reduce 443 × 463 × 1 Pixels to 84 × 84 × 1 Pixels by resizing.

### 3.2. Reward Function

**Figure 5:**
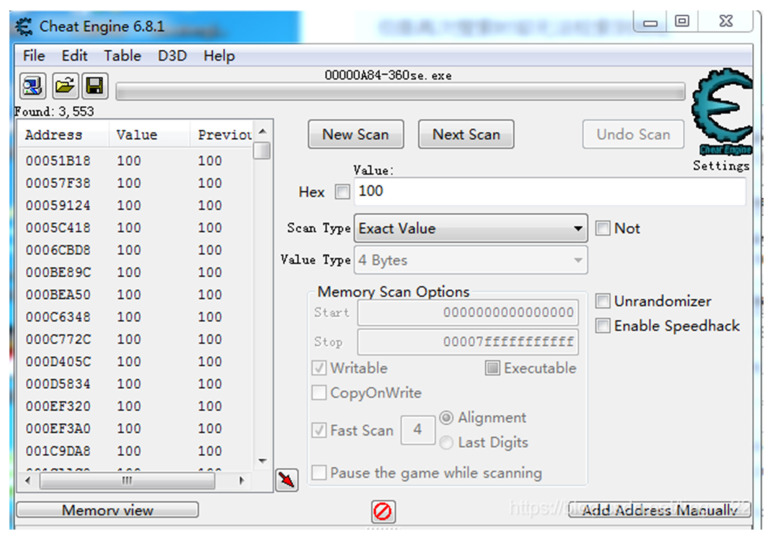
Cheat Engine.

**Figure 6:**
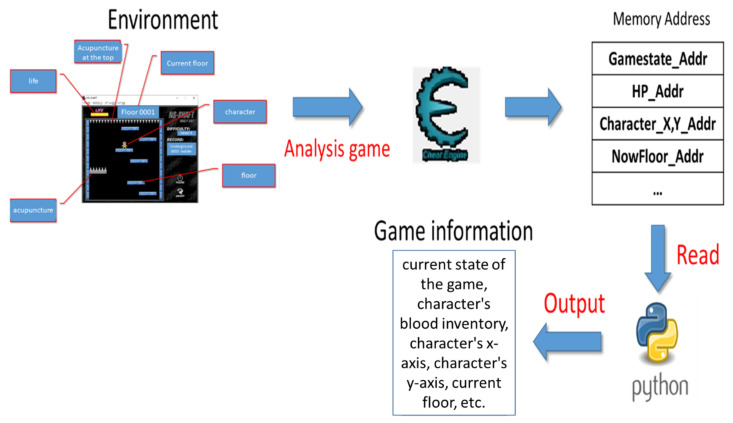
The flowchart of obtaining game internal information.

Since NS-SHAFT does not provide any way to directly obtain game internal information shown in Table 3 and does not provide any game information API, we use open source software, Cheat Engine [30], to analyze the information to obtain relevant numerical memory addresses and required game information of Reward Function to achieve real-time training for NS-SHAFT. As shown in Figure 5, Cheat Engine can modify or view the memory of the program after de-assembly. Moreover, it also locates the relevant values in the game and then achieve the operation of the overall experimental platform and the calculation of Reward. The obtained relevant numerical memory addresses and required game information of Reward Function are loaded by API of Python. The flowchart is shown in Figure 6.

In this paper, we use the current state of the game, character’s blood inventory, character’s *y*-axis, and current floor to design the Reward Function as follows. We read the game information to make judgments after each action. Since the death of the character will lead to the end of the game, we hope that the character can avoid performing the action that causes the death. If the action is taken, the agent will be killed. People (Agent) will receive negative rewards, and the cause of death has two important factors: the character’s blood volume and the character’s *y*-axis. In terms of the character’s blood volume, in order to prevent the character from encountering a needle, the state and action are calculated After the state (S’) the difference in blood volume between the two states. If the value is negative, it means that a needle stick after the action will reduce the blood volume and will be given a negative reward. In terms of the character *y*-axis, we expect the character to be in a suitable interval for the game to proceed smoothly. If it is not in this interval, then Give a negative reward, otherwise, give a positive reward. The most intuitive evaluation value in the final game is the current floor number. We recorded the number of floors before and after the action. If the two are subtracted and the number is positive, it means that the floor has been raised after the action. We will give positive rewards to encourage agents to learn the strategy.

### 3.3. Neural Network Architecture Used by DQN

For processing game screen and predicting action, we respectively adopt the Neural Network architecture used by DQN. First of all, the preprocessed first four consecutive frames form a state as the input of the model to reflect the action of the character moving to the left or to the right. The input is a game screen of size 84 × 84 × 4, the first layer of convolutional layer is composed of 32 8 × 8 filters with stride of 4, the second layer of convolutional layer is composed of 64 filters of size 4 × 4 and stride of 2, and the third convolutional layer is composed of It consists of 64 filters with a size of 3 × 3 and a stride of 1. The parameter settings are shown in Table 4.

After the convolution processing is completed, it is unfolded and connected to a fully connected layer with 512 neurons, and the final output is the *Q* value of the three actions. The relevant training parameters in DQN are described in Table 5, and the optimizer (Optimizer) used when training the network is Adam, and the loss function (Loss function) uses Mean-Square Error (MSE, Mean-Square Error) as the evaluation.

## 4. Experimental Results

The experiment in this paper will carry out seven kinds of comparative experiments. First, we test the performance of DQN and DQN with Target network, and then choose DQN with Target network as our method to test Sample frequency, Frame skipping, Mini-batch size, Target network Update period, discount value, and Reward function. We also show the significance of the trained neural network estimates on the game screen. In all experiments, the degree of difficulty is fixed to the most difficult level for experimentation.

### 4.1. Experimental Environment

Hardware:

PC with CPU I7-7700 3.60 GHz, memory 32 GB, and Graphics card NVDIA GTX 1050 Ti.

Software:

PC Windows 10 (64 bits), Python, Pycharm IDE, and Tensorflow + Keras.

### 4.2. Performance Comparison between DQN and DQN with Target Network

**Figure 7:**
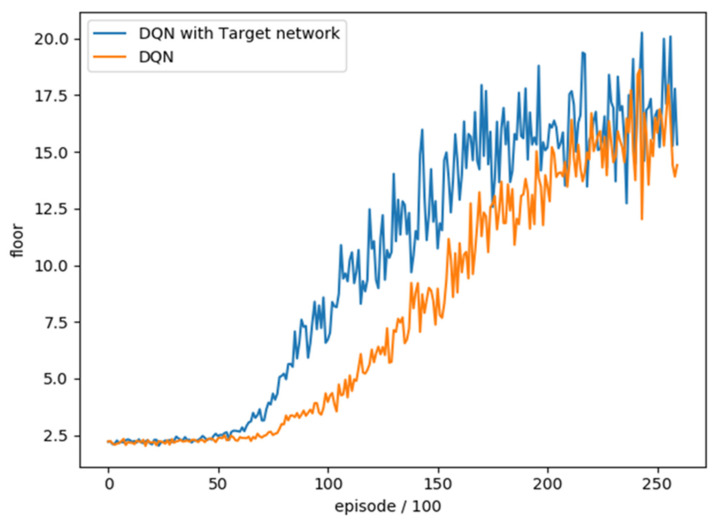
Comparison of the number of floors between DQN and DQN with Target network.

In order to understand whether the method with Target network has any difference in overall training, we trained DQN and DQN with Target network 26,000 rounds each and combined with the average floor reached every 100 rounds as an evaluation. The training trend is shown in Figure 7. We can see that the difference between the two is the growth rate of training effectiveness. The method with Target network is higher than the original DQN at about 10,000 training. We also compared the average number of reached floors in the best 100 rounds. As shown in Table 6, the results show that DQN with Target network is relatively good. Therefore, we choose DQN with Target network as our method.

### 4.3. Effect of Sample Frequency

**Figure 8:**
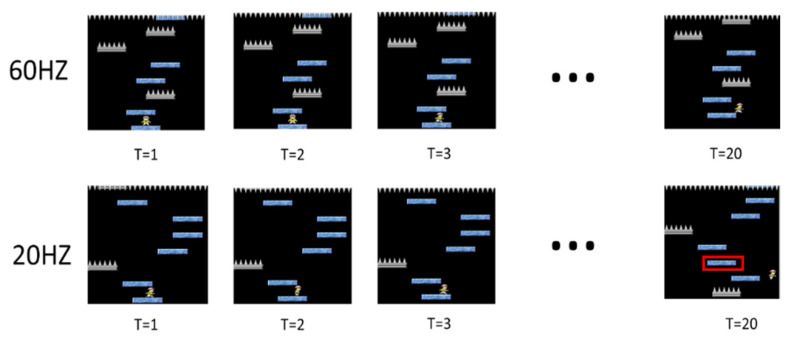
Effect of sample frequency.

Sample frequency refers to how many pictures need to be captured per second for training, and the amount of sample frequency affects the amount of picture change. The schematic diagram is as shown in Figure 8.

Taking 60 pictures per second, we can see that there is almost no change in the pictures at the three time points of T = 1~3. If taking 20 pictures per second, we can clearly see that the characters in the picture have been done. The action of moving to the right is now, and the difference is even greater at T = 20. The 60 Hz Sample frequency is about to leave the original floor, while the 20 Hz Sample frequency has already left the original floor, and the picture has changed. Then you can see the next floor with acupuncture.

In order to understand whether different Sample frequencies have an impact on the training of DQN with Target network, we use sample frequency as the test item and the rest of the parameters are unchanged, as shown in Table 7.

**Figure 9:**
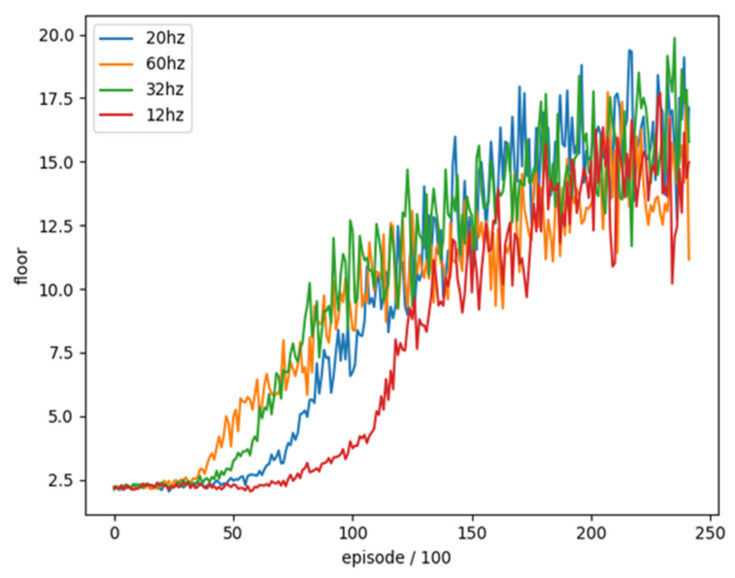
The training trend of sample frequency.

We trained these four different Sample frequencies separately for 24,200 rounds and combined with the average floor reached every 100 rounds as an evaluation. The training trend is shown in Figure 9.

We can see that the results of training at a too low sample frequency (12 Hz) are much slower than others and cannot achieve a good result in the end. Although the sample frequency (60 Hz) is too high at the beginning, the training effect has a better trend than others, but it is ultimately lower than other sample frequencies. We compared the best 100 rounds of average reaching floors as shown in Table 8. The results show that sample frequency (20 Hz) is a relatively good setting, so we choose sample frequency (20 Hz) as the final training parameter.

### 4.4. Frame Skipping

Since we usually don’t take many actions in one second when playing a game, DQN does not calculate the Q value per frame, but predicts once every k frames to reduce the computational cost and collect more experience, which is called frame skipping. However, since different games may have different suitable methods and the NS-SHAFT game is more complicated, we re-evaluate whether to use frame skipping. Before the re-evaluation, we introduce the method with or without frame skipping separately as follows.

**Figure 10:**
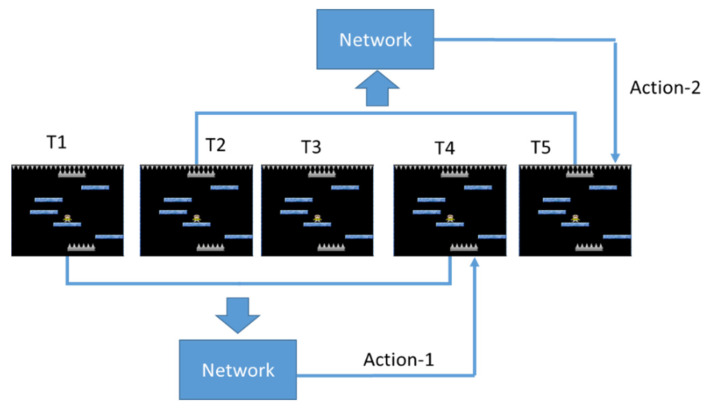
The case without frame skipping.

Figure 10 shows the case where frame skipping is not used. The game screens at the four time points of T = 1~4 are used as the input of Neural Network and predict an action. When T = 5, the screens of T = 2~5 are used as the input of Neural Network again to predict the next action.

**Figure 11:**
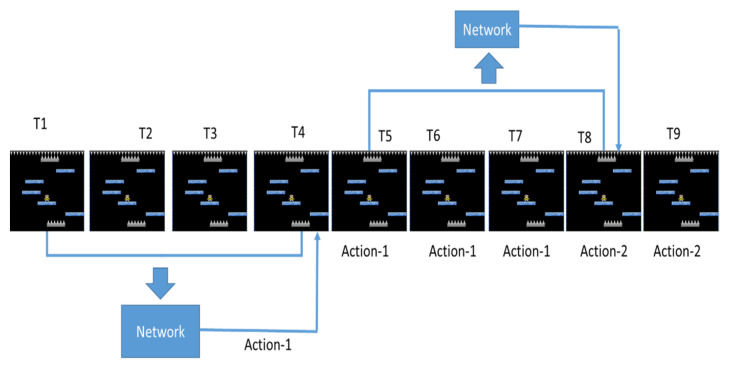
The case with frame skipping.

Figure 11 shows the use of frame skipping. The game screens at the four time points of T = 1~4 are used as the input of Neural Network, and predict an action. However, the three time points of T = 5~7 continue to use the action adopted at T = 4. Until T = 8, the screens of T = 5~8 are used as the input of Neural Network to predict the next action.

**Figure 12:**
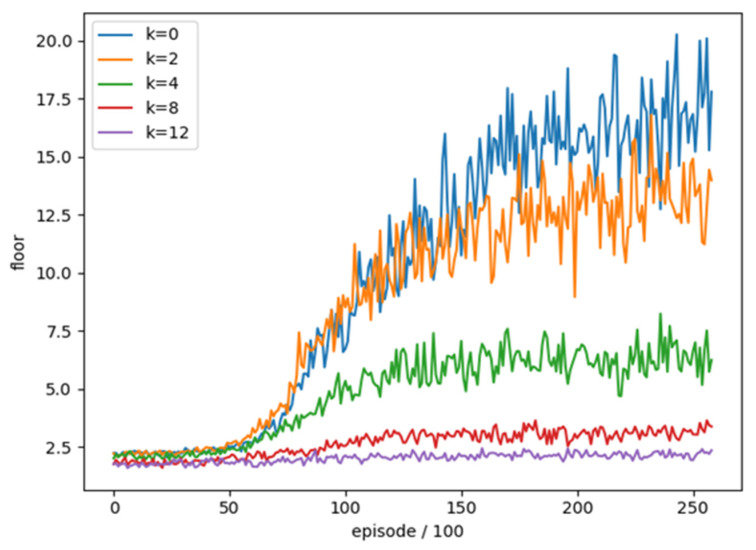
The training trend of frame skipping in different setting.

In order to understand whether the different frame skipping settings have an impact on the training of DQN with target network, we use frame skipping as test item and the rest of the parameters are unchanged for the comparison of different frame skipping settings, as shown in Table 9. We train four different frame skipping settings and compared them without frame skipping. A total of 25,900 rounds of training were combined and the average floor reached every 100 rounds was used as an evaluation. The training trend is shown in the Figure 12.

One can see that the effectiveness of training in frame skipping (4, 8, 12) is much lower than the others and cannot achieve a good result in the end. The effectiveness is the best without using frame skipping (0). The average number of reached floors in the best 100 rounds is shown in Table 10. The results also show that it is better to not use frame skipping in this experiment. Accordingly, we do not use frame skipping.

### 4.5. Mini-Batch Size

**Figure 13:**
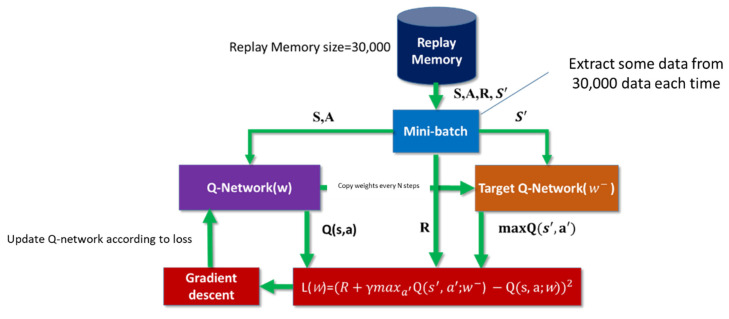
Mini-batch size.

We update the Neural Network by using Mini-batch from Replay Memory to solve the problem of high correlation of samples obtained by reinforcement learning, as shown in the Figure 13.

In order to understand whether the different settings of the Mini-batch size (number of samples taken) have an impact on the training of DQN target network, we conducted a training comparison of each mini-batch size. We used the Mini-batch size as the test item and the rest of the parameters were unchanged. The training parameters are shown in Table 11.

**Figure 14:**
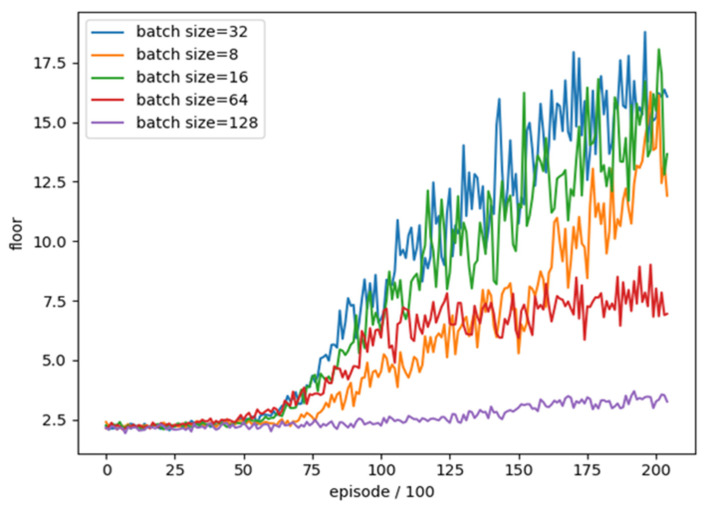
The training trend of Mini-batch size in different setting.

We trained five different batch size settings, and trained a total of 20,500 rounds, combined with the average floor reached every 100 rounds as an evaluation. The training trend is shown in Figure 14.

We can see that the effectiveness of training with too high batch size (64, 128) is much lower than others and cannot achieve a good result in the end, while the amplitude of vibration during training with too low batch size (8) is compared others are larger. Average number of reached floors in the best 100 rounds for different frame skipping settings is shown in Table 12. The results show that the moderate batch size (32) in this experiment is a better setting, so we use batch size (32) as the final training parameter.

### 4.6. Update Period of Target Network

In order to understand whether the setting of the target network update period has an impact on the DQN training, we use the target network update period as the test item and the remaining parameters are unchanged to compare the training of different target network update periods. The training parameters are shown in Table 13.

**Figure 15:**
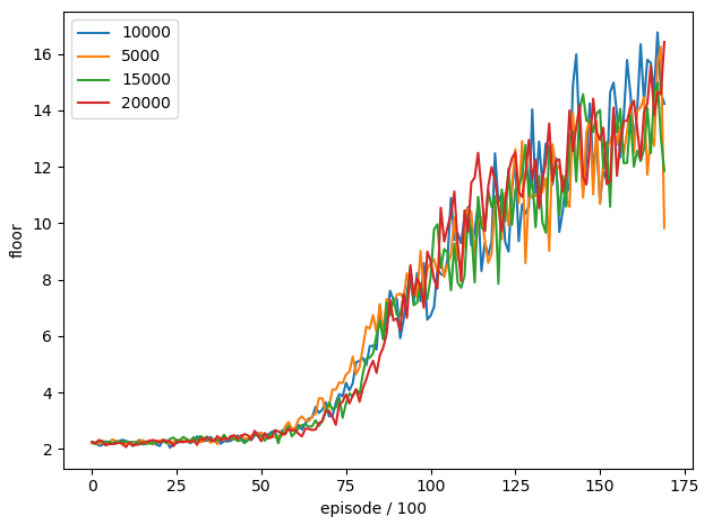
The training trend of target network update period.

We train four different Target network Update periods separately, and train 17,100 rounds in total. The average floor is reached every 100 rounds as an evaluation. The training trend is shown in Figure 15.

We can see that there is no significant difference in the comparison of the Target network Update period. It may be necessary to set the parameter to be larger to have a significant difference. Average number of reached floors in the best 100 rounds for different target network update periods is shown in Table 14. Target network update period (10,000) is a better setting, so we use Target network update period (10,000) as the final training parameter.

### 4.7. Discount Value

When DQN calculates the target value, it will multiply the future maximum action value $\gamma \underset{a^{\prime }}{\max}Q(s^{\prime },a^{\prime })$ by a discount value (*γ*), which means that we value the benefits that can be brought to us in the future when the discount is larger; otherwise, we value the benefits of the moment without considering the future.

**Figure 16:**
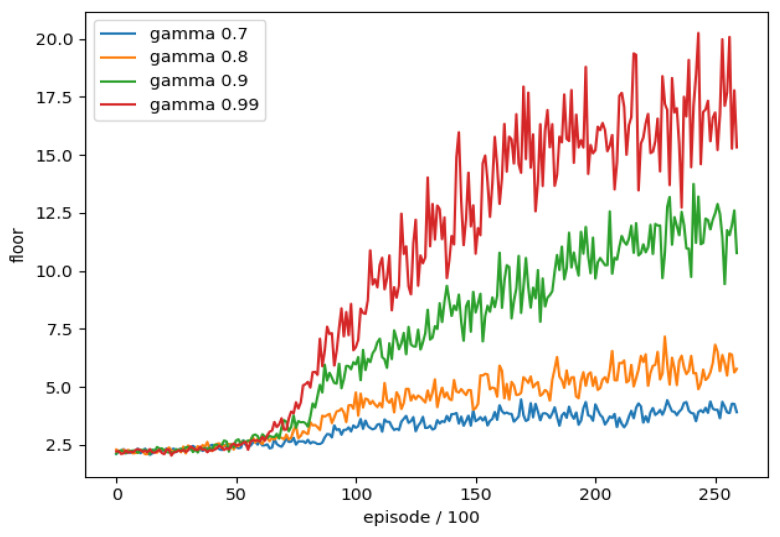
The training trend of discount value.

In order to understand whether the different discount value (γ) settings have any difference in DQN training, we conducted a training comparison of each discount value (γ). We took the discount value (γ) as the experimental item and the rest of the parameters were unchanged, and we trained separately four different discount value (γ) settings, a total of 26,000 rounds of training combined with the average floor reached every 100 rounds as an evaluation, the training trend is shown in Figure 16.

We can see that the lower discount value (0.7, 0.8) training effect is much lower than the others, and that closer (0.9, 0.99) gets better results from the higher value (0.99), so we use the discount value (0.99) is used as the final training parameter.

### 4.8. Reward Function

By the testing from 4.3 to 4.7, the parameters of DQN with target network for our architecture are determined as shown in Table 15.

After finishing the parameter setting, we will compare the design of different reward. There are two ways to define the target value y in DQN. First, it is judged that if an action is taken that leads to the end of the game round, the terminal is set to true, which means that the actual value after the action is only the reward obtained at the moment, and there is no maximum action reward value that can be obtained in the next state. In another case, the game continues after the action has not ended. At this time, the terminal is false, which means that the actual value after the action includes not only the reward currently obtained, but also the maximum reward value of action in the next state. We assume the content of reward function as Table 16.

**Figure 17:**
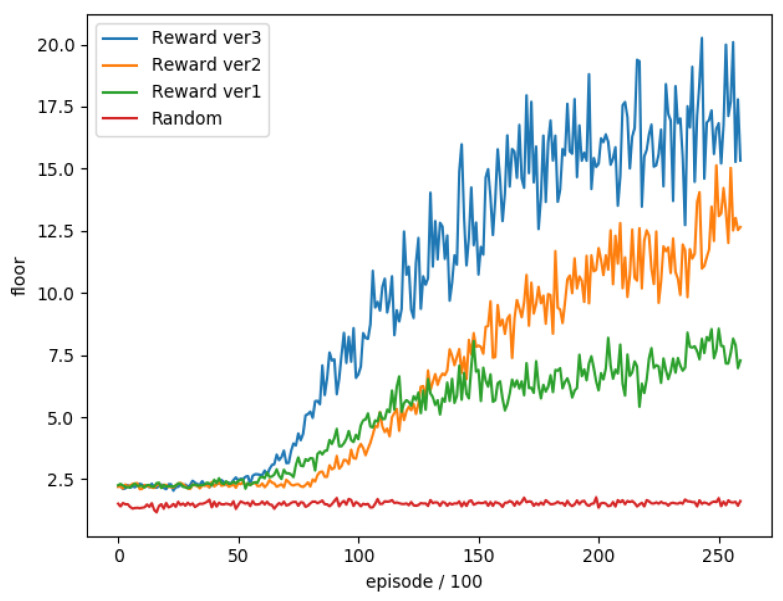
The training trend of the three versions of reward function.

Then we trained these three versions of reward separately, and compared with the completely random selection of actions, a total of 26,000 rounds were trained and the average floor reached every 100 rounds was used as an evaluation. The training trend is shown in Figure 17.

We can see that the performance of version 3 in training greatly exceeds the performance of other versions. Set terminal to true when the character loses blood, so that the neural network avoids blood loss actions, thereby increasing the possibility of survival for the game. Continue, and under the comparison of positive rewards, we found that version 1 only rewards when the floor increases after the action. It is too sparse for the neural network to effectively improve the overall performance, so we set the character *Y* axis value in the appropriate range It is correct to give positive rewards to motivate the character to stay in the proper range from time to time.

We use the ε-greedy strategy for action selection during training, and we set ε to eventually drop to 0.1 and remain unchanged, which means that every time an action is selected, there will be a 10% chance of randomly selecting the action output, in order to verify whether the model meets the training results; therefore, we will apply each trained model to the game for verification, and select the maximum Q value action as the output each time.

**Figure 18:**
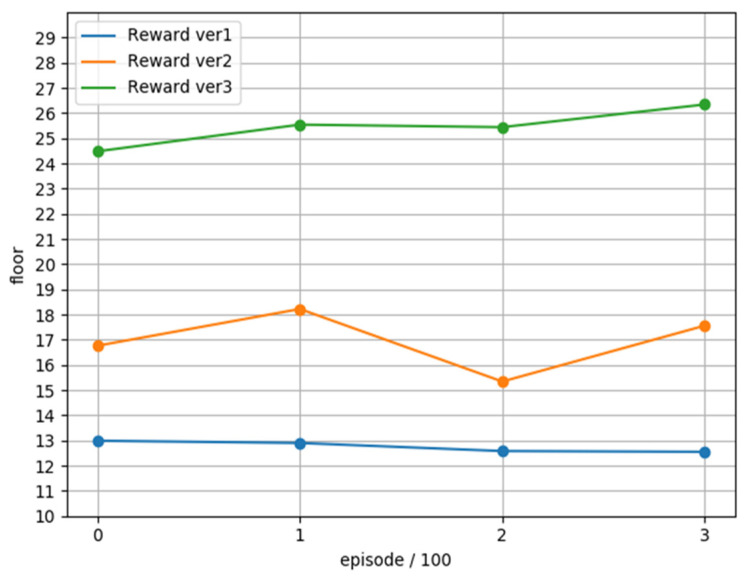
Verify the results of 400 rounds separately.

We tested the three versions of the model for 400 rounds, and plotted the average of each 100 rounds into Figure 18. It can be found that the average achieved floor of each version is higher than the value achieved during training, which can confirm that each model can be applied the game, and we found that the average achieved floors of version 3 are all above 20 floors, while version 1 is below 15 floors, which once again confirms how to define the reward function is an important key in reinforcement learning.

Finally, we counted the highest reached floors and the highest 100 average floor information in 400 rounds of each version, as shown in Figure 18.

We tested the three versions of the model for 400 rounds respectively, and plotted the average of each 100 rounds into the above figure. We can find that the average achieved floor of each version is higher than the value achieved during training, which can confirm that each model can be applied the game, and we found that the average achieved floors of version 3 are all above 20 floors, while version 1 is below 15 floors, which once again confirms how to define the reward function is an important key in reinforcement learning.

**Figure 19:**
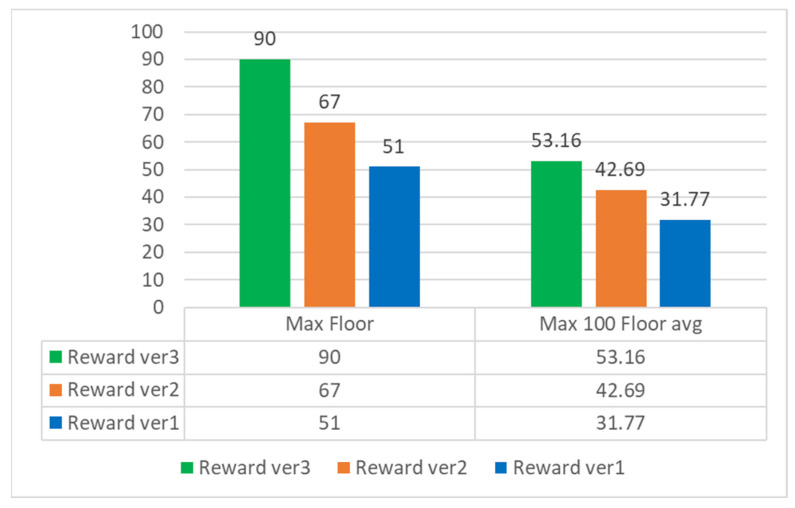
400 round statistics.

Finally, we counted the highest reached floor and the highest 100 average floor information in 400 rounds of each version, as shown in Figure 19.

**Figure 20:**
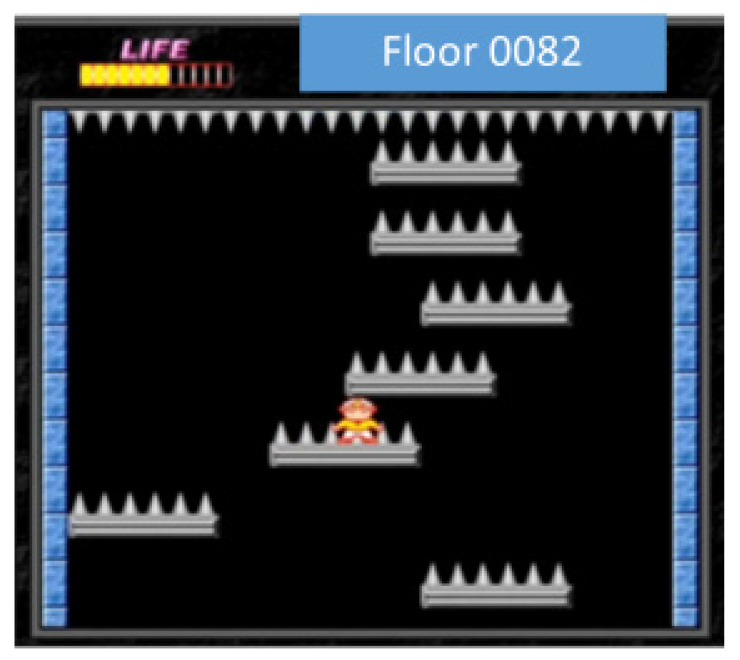
80-layer schematic.

In 400 rounds, the highest floor of version 3 reached 90. The number of needles in the game increased sharply after the 80th floor, as shown in Figure 20, so we can regard the 80th floor as the end of the game, and the average of the highest 100 pens reached 53 layers, it can be seen that in a few cases of this game, version 3 can reach the level of ordinary players or even surpass some players.

### 4.9. Q value Visualization

**Figure 21:**
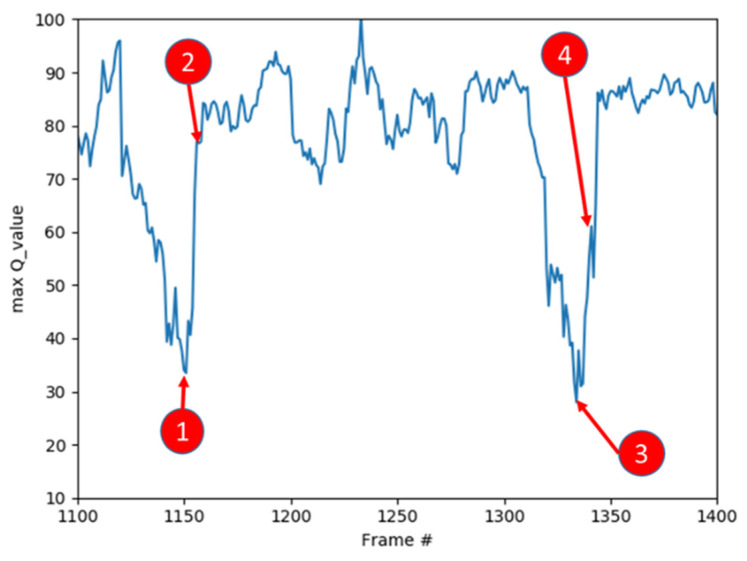
Trend graph of maximum *Q* value. Each meaning of numbers 1–4 is explained in Figure 22.

After training the model, in order to understand the meaning of the maximum action Q value estimated by the neural network in the game screen, we observed a period of the game. The trend of the maximum Q value is shown in Figure 21.

**Figure 22:**
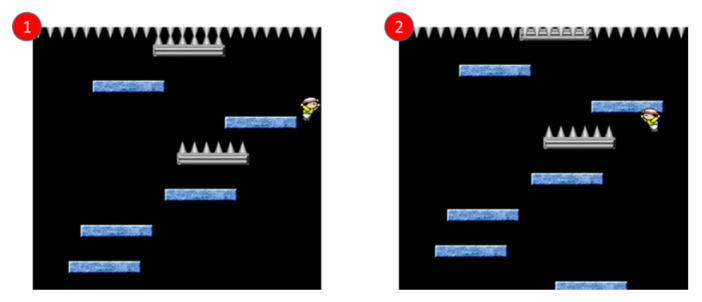

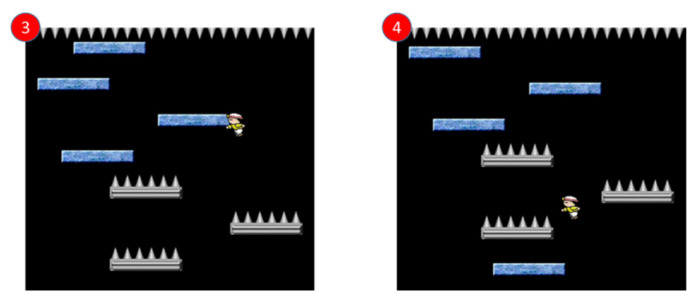
Actual game screen marked 1, 2, 3, and 4 in the trend graph of maximum Q value.

We recorded the maximum action *Q* value estimated by the neural network from the 1100th frame to the 1400th frame in the game. As shown in Figure 22, we will use the game screen in which the Q values of the two parts of the trend graph increase from low to high for explanation.

First, we see the game screen marked 1 and we can find that the character in this state is in a very bad position, and there is no floor below to keep the character alive. At this time, the Q values given by the network to the three actions are: Still: 30, Move right: 33, Move left: 31, no matter what action is taken, it is not good for the game to continue. Then, we see that the game screen marked 2 and we can find that there is a normal floor under the character. If the character is at this point, it can continue the game, the Q values given to the three actions by the network at this time are: static: 65, moving right: 63, moving left: 67. Taking a leftward action is most beneficial to land on the floor, so the network The highest estimate is given for the movement to the left.

When we see that the game screen is marked 3, we can find that the character in this state is in a very bad position. The needles below will cause the character to reduce the blood volume. At this time, the Q value given by the network for the three actions are: static: 27. Move to the right: 25. Move to the left: 28. No matter what action is taken, it is not good for the game to continue. Then, when you see the game screen marked 4, you can find that there is a normal floor under the character. If the character moves to the left, it will If there is a chance at this point, the Q values given to the three actions by the network at this time are: static: 55, moving right: 39, moving left: 61, taking a leftward action is most beneficial to land on the floor, so the network gives the highest estimate of the movement to the left.

By analyzing the Q value predicted by the Neural Network one by one, we can find that the Neural Network we trained gives the correct estimation in each state of the game and clearly understands the actual meaning of its Q value in the game.

Finally, we give a comparison with other methods by the average number of reached floors in the best 100 rounds of 2600 rounds. From the results shown in Table 17, the performance of the proposed method is better than the methods in Sarsa [13] and HNeat Pixel [18]. When comparing with the methods proposed in HNeat Best [18], DQN [14], and DQN best [15], the proposed method shows comparable performance.

## 5. Conclusions

In this paper, we successfully used deep reinforcement learning technology in the real-time game environment, and built a real-time game operation learning system with Cheat Engine as API. From the experimental results, it is still a bit behind the average performance of the average player. When comparing with the architecture that takes completely random actions, our method has taken a correct step for this game. Moreover, we found that the performance is affected by low parameter value or high parameter value. We also compared the settings of different reward functions and found that the definition of terminal and the internal information of the game can effectively improve the overall training results.

## Figures and Tables

**Table 1:** *Q*-table.

*Q*-Table		Actions			
		↑	↓	←	→
State	0	1	0	3	8
	1	−2.1	2	2.2	1
	2	4.66	5	4.9	1.23
	3	7	1	6	7.6
	4	0	2	1.1	0.6
	5	4.2	9.12	5.12	3.2
	…	…	…	…	…

**Table 2:** The comparison results of applying DQN to Atari game.

	B.Rider	Breakout	Enduro	Pong	Q*bert	Seaquest	S.Invaders
Random	354	1.2	0	−20.4	157	110	179
Sarsa [13]	996	5.2	129	−19	614	665	271
Human battle	7456	31	368	−3	18,900	28,010	3690
HNeat Best [19]	3616	52	106	19	1800	920	1720
HNeat Pixel [19]	1332	4	91	−16	1325	800	1145
DQN [14]	4092	168	470	20	1952	1705	581
DQN best [15]	5184	225	661	21	1740	1740	1075

**Table 3:** Game internal information.

Information Item	Value	Description
The current state of the game	0	The character is alive and game runs normally.
	1	The character dies, waiting for the game to start.
	2	Character is dead.
	3	The game is paused.
Game difficulty	1~3	1: simple 2: normal 3: difficult
Character’s blood inventory	0~12	If the character’s blood inventory is equal to 0, the character dies and the game ends.
Character’s *x*-axis	0~352	The movable range of the character on the *x* axis.
Character’s *y*-axis	0~352	The movable range of the character on the *y* axis. If the moving range of the character exceeds 352, it will die and the game will end.
Current floor	1~9999	Current number of floors
Acupuncture deduction	5	Blood deduction by acupuncture

**Table 4:** Parameter of convolutional layer.

	Filters	Filter Size	Activation	Stride
Convolution2D	32	8 × 8	ReLU	4
Convolution2D	64	4 x 4	ReLU	2
Convolution2D	64	3 x 3	ReLU	1

**Table 5:** Parameter of DQN.

Parameters	Description
Mini-batch size	Number of randomly selected training samples
Replay memory size	The number of records stored in the experience pool, and the oldest data will be removed when exceeded
Update period	How many actions are performed and then a training session
Learning rate	Adjust the speed of neural network weights
ε-greedy initial value	Initial value of Epsilon
ε-greedy final value	Final value of Epsilon
Final exploration frame	The number of frames required for Epsilon to fall from the initial value to the final value
Replay start size	the required size of the experience pool when starting training
Target network Update period	How many steps does the Target network update to the weight of the *Q* network
Frame skip	How many frames to predict an action
Sample frequency	How many game screens are sampled in 1 s

**Table 6:** Average number of reached floors in the best 100 rounds.

Method	Average Number of Reached Floors in the Best 100 Rounds
DQN	18.63
DQN with Target network	20.26

**Table 7:** The parameters for testing sample frequency.

Parameter	Value
Mini-batch size	32
Replay memory size	30,000
Update period	4
Learning rate	0.00001
*ε*-greedy initial value	1
*ε*-greedy final value	0.1
Final exploration frame	1,000,000
Replay start size	1000
Target network update period	10,000
Frame skipping	0
Sample frequency	Test item

**Table 8:** Average number of reached floors in the best 100 rounds for 4 sample frequencies.

Sample Frequency	Average Number of Reached Floors in the Best 100 Rounds
20	19.1
60	17.73
32	19.03
12	17.69

**Table 9:** The parameters for testing frame skipping.

Parameter	Value
Mini-batch size	32
Replay memory size	30,000
Update period	4
Learning rate	0.00001
*ε*-greedy initial value	1
*ε*-greedy final value	0.1
Final exploration frame	1,000,000
Replay start size	1000
Target network update period	10,000
Frame skipping	Test item
Sample frequency	20 Hz

**Table 10:** Average number of reached floors in the best 100 rounds for different frame skipping settings.

Frame Skipping Settings	Average Number of Reached Floors in the Best 100 Rounds
0	20.6
2	16.79
4	8.23
8	3.63
12	2.43

**Table 11:** The parameters for testing sample Mini-batch size.

Parameter	Value
Mini-batch size	Test item
Replay memory size	30,000
Update period	4
Learning rate	0.00001
*ε*-greedy initial value	1
*ε*-greedy final value	0.1
Final exploration frame	1,000,000
Replay start size	1000
Target network update period	10,000
Frame skipping	0
Sample frequency	20 Hz

**Table 12:** Average number of reached floors in the best 100 rounds for different frame skipping settings.

Mini-Batch Size Settings	Average Number of Reached Floors in the Best 100 Rounds
32	18.8
8	16.28
16	18.07
64	9.01
28	3.69

**Table 13:** The parameters for testing target network update period.

Parameter	Value
Mini-batch size	32
Replay memory size	30,000
Update period	4
Learning rate	0.00001
*ε*-greedy initial value	1
*ε*-greedy final value	0.1
Final exploration frame	1,000,000
Replay start size	1000
Target network update period	Test item
Frame skipping	0
Sample frequency	20 Hz

**Table 14:** Average number of reached floors in the best 100 rounds for different target network update period.

Target Network Update Period	Average Number of Reached Floors in the Best 100 Rounds
5000	16.76
10,000	16.25
15,000	14.97
20,000	16.42

**Table 15:** The parameters setting of DQN with target network for our architecture.

Parameters	Value	Unit
Mini-batch size	32	transition
Replay memory size	30,000	transition
Update period	4	frame
Learning rate	0.00001	
*ε*-greedy initial value	1	
*ε*-greedy final value	0.1	
Final exploration frame	1,000,000	transition
Replay start size	1,000	frame
Target network update period	10,000	frame
Frame skipping	0	frame
Sample frequency	20Hz	frame

**Table 16:** The content of reward function.

Version 1	After the action, if the floor increases, a positive reward (+1) will be given. If the character dies after the action, a negative reward (−1) will be given. If the character’s blood inventory decreases after the action, a negative reward (−1) will be given.
Version 2	After the action, if the character’s *Y*-axis value is not within the defined range (100, 260), a negative reward (−3) will be given. If the character dies after the action, a negative reward (−10) will be given. If the character’s blood volume decreases after the action, a negative reward (−5). Positive rewards (+1) for other states.
Version 3	The content is roughly the same as that of version 2. The difference lies in the definition of terminal. In version 1 and version 2, terminal is set to true only when the character dies (representing the end of the game round), while version 3 sets the character’s HP after the action The reduced state also sets terminal to true, but in fact the game is still in progress, and its impact is that the target value update calculation is different.

**Table 17:** The comparison with other methods by the average number of reached floors in the best 100 rounds of 2600 rounds.

	Average Floors (Score) of NS-SHAFT Game
Sarsa [13]	7.86
HNeat Best [18]	17.54
HNeat Pixel [18]	9.25
DQN [14]	18.63
DQN best [15]	20.26
The proposed method	20.31

## Data Availability

Not applicable.
